# Thin therapeutic endoscope combination surgery based clip closure for mucosal defect after colorectal endoscopic submucosal dissection

**DOI:** 10.1055/a-2589-1763

**Published:** 2025-05-22

**Authors:** Takuma Okamura, Tomonari Ikeda, Miruki Yoshino, Tatsuki Ichikawa, Hisamitsu Miyaaki

**Affiliations:** 1 13650 Department of Gastroenterology, Nagasaki Harbor Medical Center, Nagasaki, Japan; 2 200674 Department of Comprehensive Community Care Systems, Graduate School of Biomedical Sciences, Nagasaki University, Nagasaki, Japan; 3 200674 Department of Gastroenterology and Hepatology, Graduate School of Biomedical Sciences, Nagasaki University, Nagasaki, Japan

Recently, mucosal defect closure after endoscopic submucosal dissection (ESD) has garnered attention. Several techniques have been reported as effective, including the “origami” clip method 1 , MANTIS 2 , ROLM 3 , and endoscopic hand suturing (EHS).

At our hospital, we have introduced a technique called “thin therapeutic endoscope combination surgery (TECS)” for the treatment of gastrointestinal tumors, which involves the use of two thin therapeutic endoscopes and two endoscopists, and we have reported on its various applications 4 ( Fig. 1 ). TECS allows for both resection and mucosal defect closure. This report presents a case where mucosal defect closure was performed after colorectum ESD using two endoscopes.

**Fig. 1:**
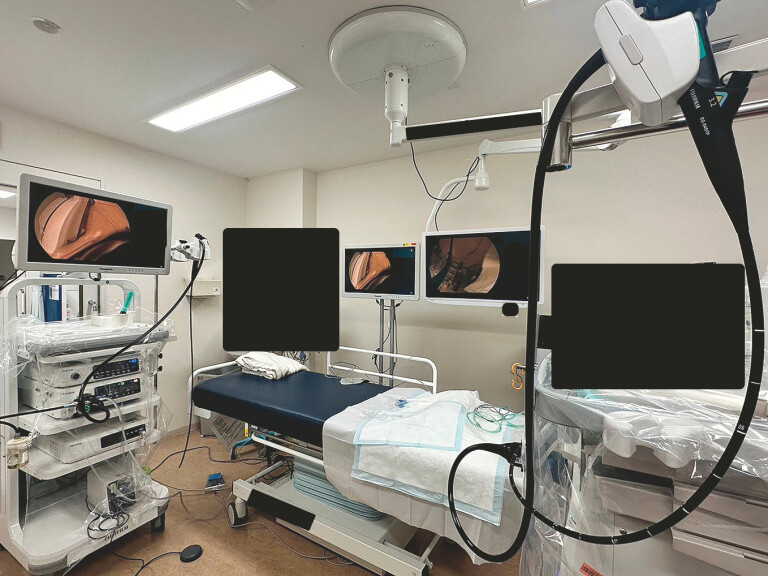
Layout of treatment rooms.

A 20 mm depressed lesion in the ascending colon was resected in 17 minutes using TECS, and the mucosal defect was 35 mm × 30 mm. ( Fig. 2 ) Mucosal defect closure was performed using a TECS-based clip closure ( Video 1 ). The operator used SureClip Plus (Micro-Tech Co. Ltd.) to grasp and elevate the muscular layer of the mucosal defect while folding it. The assistant then used SureClip Eco to secure the mucosa, muscularis, and mucosa layers, ensuring proper closure. This process was repeated multiple times. After achieving partial closure, the operator and assistant alternated applying additional clips to reduce closure time. Complete closure was achieved within 19 minutes ( Fig. 3 ).

**Fig. 2:**
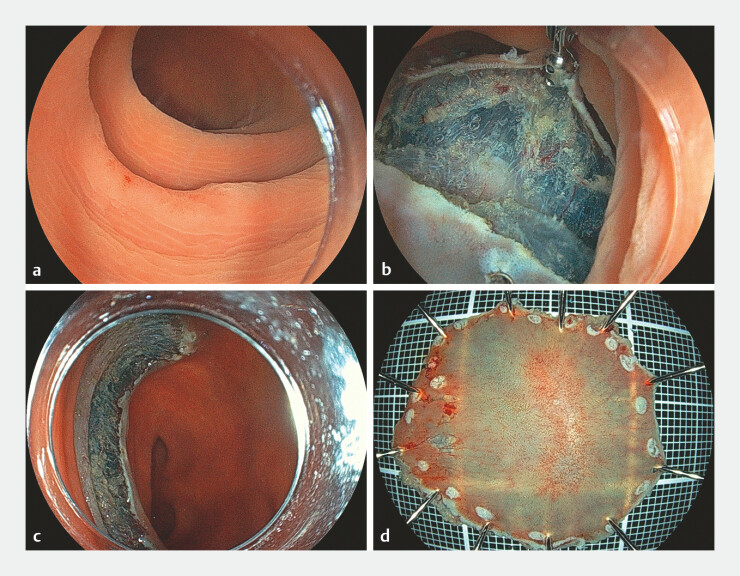
Endoscopic view of tumor and resection by TECS. **a** White-light image of a tumor; **b–c** resection with TECS; **d** The resected specimen measured 35 × 30 mm. Abbreviation: TECS, thin therapeutic endoscope combination surgery.

**Fig. 3:**
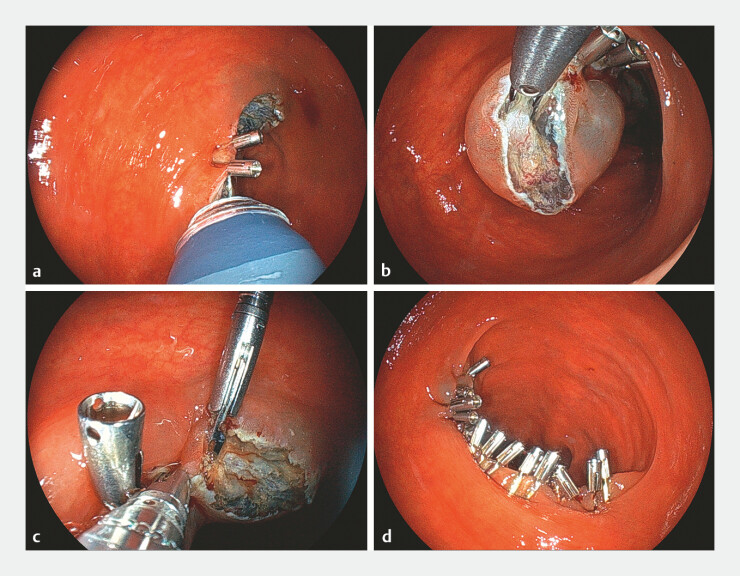
Endoscopic view of clip closure with thin therapeutic endoscope combination surgery. **a, b** The operator used the SureClip Long to grasp and lift the muscular layer. The assistant then applied a SureClip Eco to grasp the mucosa-muscularis mucosa and to secure closure. Complete closure of the mucosal defect was achieved within 19 min.

Clip closure of the mucosal defect using thin therapeutic endoscope combination surgery (TECS).Video 1

The TECS-based clip closure technique allows for dead space reduction by elevating and folding the muscular layer. While verifying proper grasping of the oral side mucosa during colonic clip closure can be challenging, adjusting the orientation of the captured mucosa simplifies the closure process and allows for easier clip application. This method utilizes conventional clips commonly used in endoscopic procedures, avoiding additional costs. Given these advantages, TECS-based clip closure is considered to be an effective approach for sealing mucosal defects following colonic ESD.

Endoscopy_UCTN_Code_TTT_1AQ_2AD_3AD

## Notes

Endoscopy E-Videos https://eref.thieme.de/e-videos
*E-Videos* is an open access online section of the journal *Endoscopy* , reporting on interesting cases and new techniques in gastroenterological endoscopy. All papers include a high-quality video and are published with a Creative Commons CC-BY license. Endoscopy E-Videos qualify for HINARI discounts and waivers and eligibility is automatically checked during the submission process. We grant 100% waivers to articles whose corresponding authors are based in Group A countries and 50% waivers to those who are based in Group B countries as classified by Research4Life (see: https://www.research4life.org/access/eligibility/ ). This section has its own submission website at https://mc.manuscriptcentral.com/e-videos .
